# Chronic Exposure to Androgenic-Anabolic Steroids Exacerbates Axonal Injury and Microgliosis in the CHIMERA Mouse Model of Repetitive Concussion

**DOI:** 10.1371/journal.pone.0146540

**Published:** 2016-01-19

**Authors:** Dhananjay R. Namjoshi, Wai Hang Cheng, Michael Carr, Kris M. Martens, Shahab Zareyan, Anna Wilkinson, Kurt A. McInnes, Peter A. Cripton, Cheryl L. Wellington

**Affiliations:** 1 Department of Pathology and Laboratory Medicine, Djavad Mowafaghian Centre for Brain Health, University of British Columbia, Vancouver, Canada; 2 Department of Biomechanical Engineering, University of British Columbia, Vancouver, Canada; 3 International Collaboration on Repair Discoveries, University of British Columbia, Vancouver, Canada; Martin Luther University, GERMANY

## Abstract

Concussion is a serious health concern. Concussion in athletes is of particular interest with respect to the relationship of concussion exposure to risk of chronic traumatic encephalopathy (CTE), a neurodegenerative condition associated with altered cognitive and psychiatric functions and profound tauopathy. However, much remains to be learned about factors other than cumulative exposure that could influence concussion pathogenesis. Approximately 20% of CTE cases report a history of substance use including androgenic-anabolic steroids (AAS). How acute, chronic, or historical AAS use may affect the vulnerability of the brain to concussion is unknown. We therefore tested whether antecedent AAS exposure in young, male C57Bl/6 mice affects acute behavioral and neuropathological responses to mild traumatic brain injury (TBI) induced with the CHIMERA (Closed Head Impact Model of Engineered Rotational Acceleration) platform. Male C57Bl/6 mice received either vehicle or a cocktail of three AAS (testosterone, nandrolone and 17α-methyltestosterone) from 8–16 weeks of age. At the end of the 7^th^ week of treatment, mice underwent two closed-head TBI or sham procedures spaced 24 h apart using CHIMERA. Post-repetitive TBI (rTBI) behavior was assessed for 7 d followed by tissue collection. AAS treatment induced the expected physiological changes including increased body weight, testicular atrophy, aggression and downregulation of brain 5-HT1B receptor expression. rTBI induced behavioral deficits, widespread axonal injury and white matter microgliosis. While AAS treatment did not worsen post-rTBI behavioral changes, AAS-treated mice exhibited significantly exacerbated axonal injury and microgliosis, indicating that AAS exposure can alter neuronal and innate immune responses to concussive TBI.

## Introduction

Traumatic brain injury (TBI) is a leading worldwide cause of death and disability with a cost to society of over $76B USD per year. The global annual incidence of TBI is estimated to be approximately 200 per 100,000 persons [1]. In the United States, the overall incidence of TBI is estimated to be 538 per 100,000 persons, which represents at least 1.7 million new cases per year since 2003 [2–4]. Compounding this is the growing awareness that > 75% of TBI are mild (mTBI, a term synonymous with concussion) [2] that do not necessarily need hospitalization and therefore are not always reported. The past decade has witnessed a tremendous surge of interest in concussion in youth and young adults, as this age group represents a major peak of TBI incidence for whom decreased educational and occupational achievement could have profound long-term consequences. Concussions in youth and young adults are attributed mostly to falls, motor vehicle accidents, and participation in sports. According to the US Centers for Disease Control and Prevention estimates, emergency departments in the United States treated approximately 173,285 sport-related concussion (SRC) cases annually during 2001–2009 and approximately 71% of the SRC visits were among persons aged 10–19 years [5]. Falls account for TBI in nearly half of the persons aged 0–14 years followed by struck by/against events [4]. As current diagnosis relies on subjective symptom reporting, there is great of interest in characterizing the cellular and histopathological changes that occur after concussion to aid in the development of evidence-based objective, sensitive and specific metrics of concussion diagnosis, prognosis and recovery in young people.

Post-concussion syndrome (PCS) is a term used to describe a constellation of symptoms across emotional, somatic and cognitive domains by approximately 50% of patients with mild TBI [6, 7]. Although PCS typically resolves within 3 months of injury for the majority of patients, some develop persistent PCS [6, 7]. However, the symptoms of PCS are nonspecific and also found in patients with traumatic injuries to areas of the body other than the head [8, 9]. The nonspecificity of PCS symptoms led the International Collaboration on Mild Traumatic Brain Injury Prognosis to recommend that the term “post-concussion syndrome” be replaced with “posttraumatic symptoms PTS)” [10]. Factors that influence symptom prevalence include concurrent components such as pain, anxiety, depression, post-traumatic stress, pre-existing psychiatric conditions and litigation [11, 12]. With respect to factors that predict symptom reporting, a recent investigation of close to 32,000 uninjured high school athletes (without concussion for 6 months) found that symptom reporting was more common in girls, especially those with prior treatment of a psychiatric condition or substance abuse and attention deficit-hyperactivity disorder [13]. For boys, prior treatment of a psychiatric condition was the strongest independent predictor for symptom reporting, followed by a history of migraines [13]. Intriguingly, the weakest independent predictor of symptom reporting for both sexes was history of prior concussion [13].

Given the challenges associated with monitoring concussion recovery using subjective and nonspecific symptoms, objective and quantifiable biomarkers are highly desirable. Diffuse axonal injury (DAI) and white matter neuroinflammation are commonly found upon histopathological examination of TBI brain tissue. Deformation of white matter at the moment of traumatic injury is believed to lead to mechanical failure and calcium dependent proteolysis of the axonal cytoskeleton, leading to several markers of axonal damage that can include silver uptake as well as accumulation of amyloid precursor protein (APP), neurofilament and proteolytic fragments of alpha II spectrin [14]. Neuroinflammation is another well-recognized response to TBI in both humans and experimental models. As the activation of immune and non-immune cells that occurs in the days to weeks after injury can influence many post injury symptoms, the term Post-Inflammatory Brain Syndrome has recently been suggested [15]. Neuroinflammation is also a major component of many neurodegenerative diseases of aging, and a dysregulated neuroinflammatory response after TBI could contribute to the increased risk of chronic consequences of TBI.

One such consequence of interest to those who participate in high contact sports is chronic traumatic encephalopathy (CTE), a progressive neurodegenerative disease clinically characterized by alterations in mood and behavior, motor disturbances and, in severe cases, progressive dementia [16, 17]. Originally described as dementia pugilistica [18], recent studies have revealed that similar clinical symptoms and neuropathology are also observed in other contact sports such as American football, boxing, hockey, and association football (soccer) [19–21]. CTE cannot yet be confirmed without post mortem analyses [22] although emerging brain imaging data using [F-18]FDDNP PET may hold promise detecting tauopathy in living persons with susceptibility for CTE [23]. The primary neuropathological feature of CTE is extensive deposition of hyperphosphorylated tau [20, 24].

A recent systematic review revealed that approximately 20% of all reported pathologically confirmed CTE cases have a documented history of exposure to illicit substances including androgenic-anabolic steroids (AAS), alcohol, methamphetamine and marijuana prior to or concurrent with CTE [25]. Given that 90% of confirmed cases of CTE have been in athletes, [22] determining the effects of substances that are used by athletes on TBI outcome may provide useful information on TBI pathogenesis.

AAS are synthetic derivatives of testosterone used by both elite and recreational athletes [26, 27] for their ergogenic effects including increased muscle strength, endurance and power, increased lean body mass, and enhanced recovery between workouts and from injury [28]. A recent meta-analysis of 271 studies found the global prevalence rate of AAS use among elite and recreational athletes to be 13.4% and 18.4%, respectively, although this may be an underestimate due to the illegal nature of ASS use [29]. Further, athletes younger than 19 years have a higher prevalence of AAS (2.5%) compared to athletes aged older than 19 years (1.9%) [29]. AAS use in athletes is driven by perceived benefits to both athletic performance and appearance. While AAS may enhance sports performance, chronic AAS use is associated with several adverse psychiatric effects including increased aggression, depression, mood and anxiety disorders, irritability and suicidal tendencies [27, 30]. Chronic AAS use also induces widespread adverse physiological alterations involving multiple organ systems, particularly of the hypothalamic-pituitary-gonadal axis [31]. Moreover, preclinical and clinical studies indicate altered structural remodeling and neurotransmitter physiology in adolescent brains following AAS exposure, which also may affect behavior [32].

As several of the psychiatric effects associated with AAS use overlap with clinical symptoms of PTS, it is possible that AAS exposure may alter the brain’s response to impact. AAS-related aggressive behaviors may also increase the probability of experiencing head trauma both on and off the field. Ethical constraints clearly pose considerable challenges for controlled clinical studies of how AAS exposure affects both TBI risk and pathogenesis in athletes. To our knowledge, only one preclinical study to date has investigated the effects of a 12-week exposure to a single AAS, nandrolone, on axonal injury in rats subjected to a single weight drop TBI [33]. In this study, axonal injury as assessed with amyloid precursor protein (APP) immunohistochemistry was the sole TBI outcome examined. No alteration in axonal damage was observed after TBI in AAS-treated compared to control rats when assessed 30 d post-injury.

Given that a significant proportion of both professional and recreational athletes in high contact sports are at risk for concussion and that AAS exposure in sport may be underreported, we aimed to test whether antecedent AAS exposure affects behavior, DAI and neuroinflammation in our recently described mouse model of repetitive TBI (rTBI) called CHIMERA (Closed Head Impact Model of Engineered Rotational Acceleration). CHIMERA is designed to reliably replicate the biomechanical conditions encountered in human mTBI, namely, impact to a closed skull with free head movement after impact [34]. Here we report that chronic exposure to AAS in young, wild-type male mice exacerbates rTBI-induced DAI and microgliosis in white matter without further worsening of rTBI-induced behavioral deficits.

## Results

### AAS Treatment Increases Body and Seminal Vesicle Weights along with Testicular Atrophy

**Fig 1:**
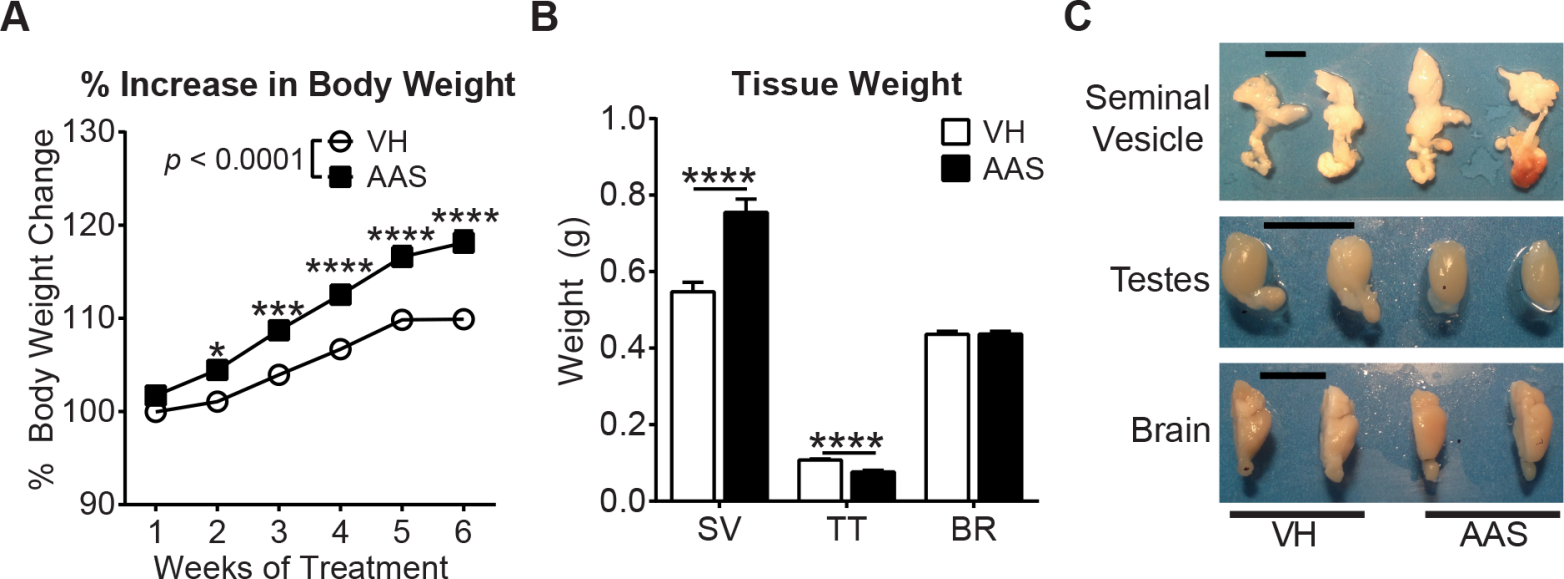
Chronic AAS treatment in mice induces physiological changes. Prior to rTBI, mice were treated 5 d per week for 7 weeks with a cocktail of androgenic-anabolic steroids (AAS) or sesame oil vehicle (VH). Cohort size: VH: N = 22, AAS: N = 23. (A) Percent increase in body weight in AAS and VH-treated mice over time. (B) Comparison of weights of seminal vesicles (SV), testes (TT), and brain (BR) collected at 7 d post-rTBI. (C) Macroscopic size comparison of seminal vesicle, testes and brain. Scale bar = 1cm. In all graphs, data are presented as mean ± SEM values. Body weight data are analyzed by two-way repeated measures ANOVA followed by a Bonferroni post hoc test, tissue weight data were analyzed by two-tailed unpaired t test. For all graphs, *: *p* < 0.05, ***: *p* < 0.001, ****: *p* < 0.0001.

We first assessed the physiological effects of chronic AAS treatment by recording body weight weekly for 6 weeks during an 8-week total treatment period. Compared to the vehicle (VH) group, mice in the AAS treatment group showed the expected persistent and significant increase in body weight starting from the 2^nd^ week following initiation of treatment through week 6 (Fig 1A, *p* < 0.0001). The body weight increase in the AAS group showed both time (*p* < 0.0001) and treatment (*p* < 0.0001) effects. Post-mortem morphological analyses of androgen-responsive tissues including the seminal vesicles and testes revealed that AAS-treated mice exhibited significantly increased seminal vesicle weight (Fig 1B, *p* < 0.0001) as well as size (Fig 1C) compared to VH controls. In addition, AAS-treated mice also showed testicular atrophy (Fig 1B and 1C) compared to VH controls. AAS treatment did not affect brain weight or size (Fig 1B and 1C).

### Chronic AAS Treatment Does Not Exacerbate Acute rTBI-Induced Behavioral Deficits

**Fig 2:**
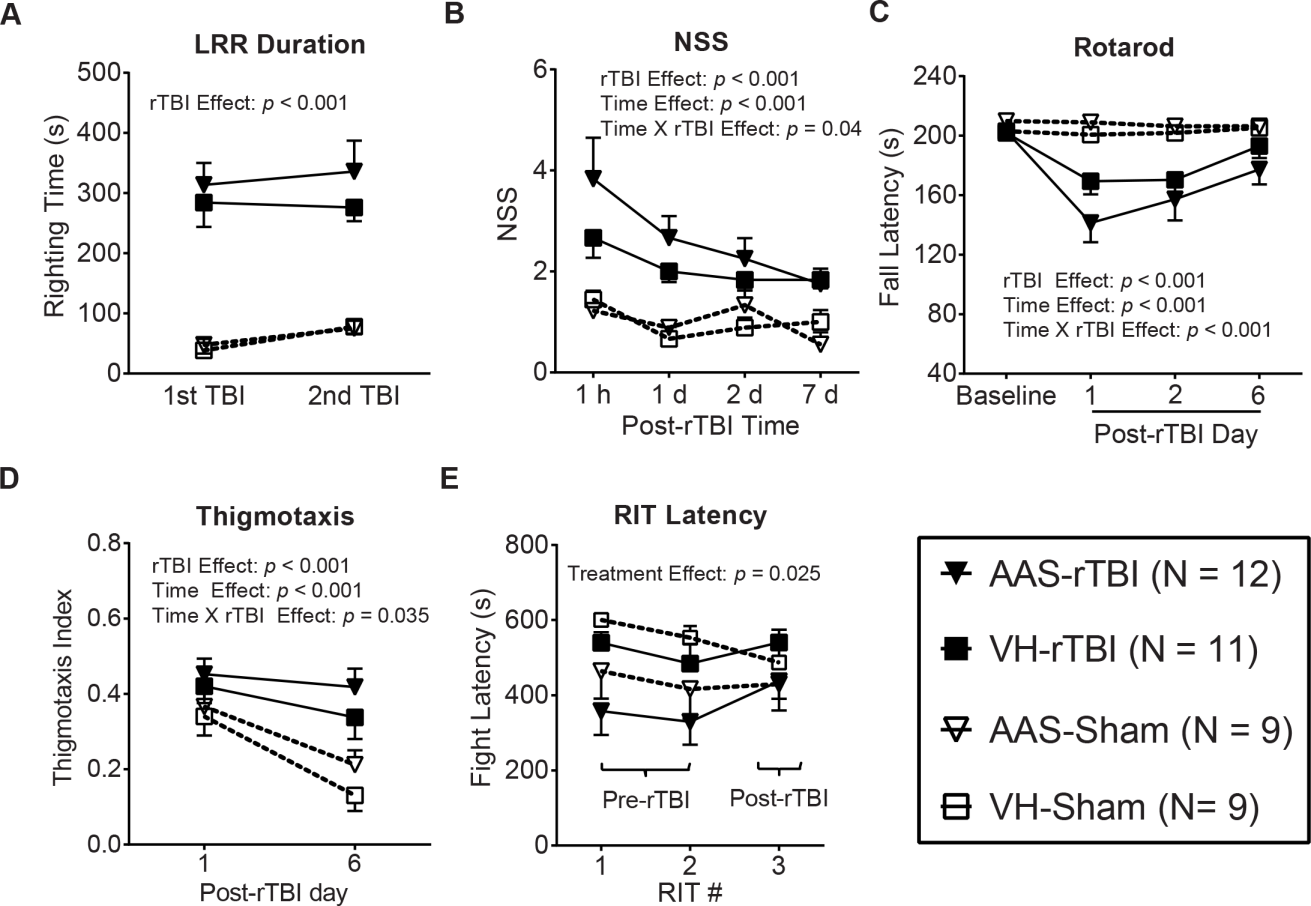
Chronic AAS treatment does not exacerbate acute post-rTBI behavioral deficits. (A) Duration of loss of righting reflex (LRR) was assessed immediately following the sham or TBI procedure. (B) Composite neurological severity score (NSS) was assessed at 1 h and at 1, 2 and 7 d post-rTBI. (C) Motor performance was assessed on an accelerating rotarod at 1, 2, and 7 d post-rTBI. The graph depicts fall latency in seconds at baseline before rTBI and at three post-rTBI time points. (D) Thigmotaxis was quantified at 1 and 6 d post-rTBI and is represented as thigmotaxis index. (E) Aggressive behavior was assessed with the RIT at the 5^th^ (RIT # 1) and 6^th^ (RIT # 2) week following initiation of AAS treatment (pre-rTBI) as well as at 5 d (RIT # 3) post-rTBI. Graphs represent latency to initiate fighting by the resident mouse. Data in all graphs are presented as mean ± SEM. Data are analyzed by repeated measures general linear model. Legends and cohort sizes are consistent across all graphs.

Mice subjected to rTBI exhibited significantly prolonged loss of righting reflex (LRR) regardless of VH or AAS treatment compared to their respective sham controls (Fig 2A, rTBI effect: *p* < 0.001). LRR duration remained stable over two impacts (time effect: *p* = 0.850) and did not show any significant treatment effect (*p* = 0.704). Injured mice in both VH and AAS groups showed significant neurological deficits as observed by higher composite neurological severity score (NSS) values compared to their respective sham controls (Fig 2B, rTBI effect: *p* < 0.001). NSS values in both injured groups were highest at 1 h post-TBI and declined over 7 d showing a significant time effect (*p* < 0.001). NSS values also showed a significant time X rTBI interaction (*p* < 0.001), indicating that NSS values of injured mice improved over time while sham animals had stable NSS scores. AAS treatment did not significantly exacerbate NSS scores in injured mice (Treatment effect: *p* = 0.352), although we noted that AAS-treated injured mice showed a trend toward higher NSS values over VH controls from 1 h to 2 d post-rTBI. A detailed analysis of individual NSS tasks revealed that 25% of mice in the AAS-rTBI group failed on the 3 cm wide beam walk test (the easiest among 1 cm, 2 cm, and 3 cm wide beam walk tasks) at 1 h post-rTBI, whereas all mice in the VH-rTBI group successfully completed the same task, albeit this finding did not reach statistical significance (S1 Fig, Fisher’s exact test, *p* = 0.217). Similarly, rTBI significantly impaired motor performance in VH-rTBI and AAS-rTBI groups as indicated by reduced fall latencies on an accelerating rotarod compared to their respective sham controls (Fig 2C, rTBI effect: *p* < 0.001). Post-rTBI motor dysfunction in both injured groups was maximal at 1 d post-rTBI. Fall latencies showed both a significant time effect (*p* < 0.001) and a significant rTBI X time interaction (*p* < 0.001), indicating that the motor performance of sham animals was stable over time but deteriorated in the rTBI groups. Although AAS treatment did not significantly affect rTBI-induced motor deficits (*p* = 0.765), mice in the AAS-rTBI group exhibited a trend toward ~ 8–16% shorter fall latencies compared to the VH-rTBI group. Injured mice showed anxiety-like behavior as indicated by significantly increased thigmotaxis in an open field test performed 6 d post-rTBI (Fig 2D, TBI effect: *p* < 0.001). Thigmotaxis showed a significant time effect (*p* < 0.001) as well as a time X rTBI interaction (*p* = 0.035). AAS treatment did not significantly alter thigmotactic behavior. Open field thigmotaxis was not affected by gross motor activity as no significant differences in total distance traveled (Injury effect: *p* = 0.619) or time immobile (Injury effect: *p* = 0.357) were observed between injured and sham-operated mice (S2 Fig). Finally, aggressive behavior was exacerbated in AAS-treated resident mice prior to injury as indicated by significantly decreased latency to fight with intruder mice as assessed by RIT at the 5^th^ and 6^th^ week of AAS treatment (Fig 2E, treatment effect: *p* < 0.05). Aggressive behavior was diminished when measured at 5 d post-rTBI (Fig 2E).

To test whether the extensive handling of the mice during the injection regimen was sufficient to alter post-rTBI behavior, we also compared the two VH treatment groups with naïve controls that were neither handled nor subjected to injections prior to sham or rTBI procedures. For all but one behavior, no differences between the VH-treated and naïve groups swere observed, indicating that the handling involved in the treatment schedule had minimal behavioral effects (S3A–S3D Fig). The single exception was that mice in the VH group exhibited hyperactivity in the open field task as shown by a significant treatment effect in the distance traveled (S3E Fig, *p* = 0.02) and immobile time (S3F Fig, *p* < 0.001).

### Neither rTBI nor Chronic AAS Treatment Affects Endogenous Murine Tau Phosphorylation 7 D Post-Injury

**Fig 3:**
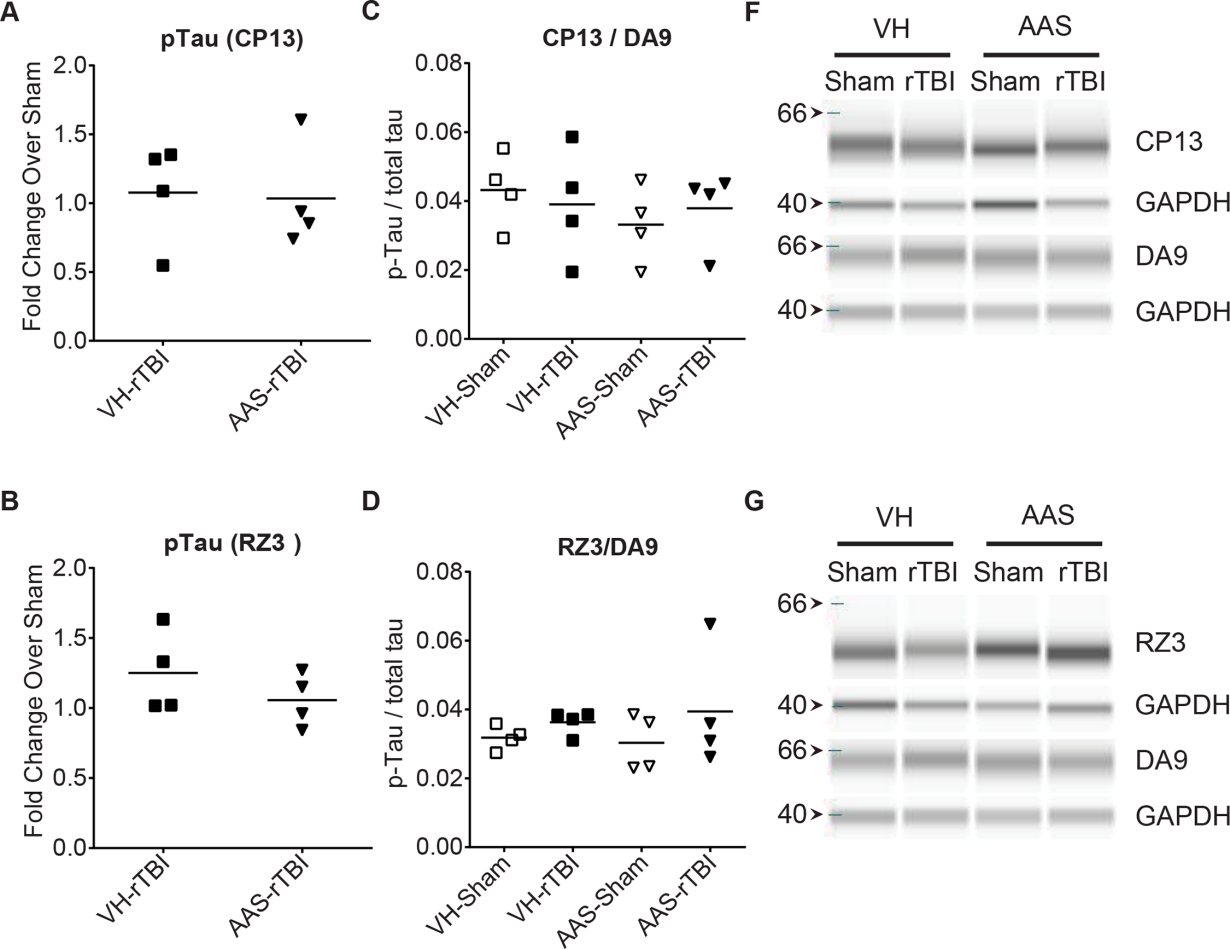
CHIMERA rTBI and AAS treatment do not affect endogenous tau phosphorylation at 7 days post injury. Tau phosphorylation was assessed using the Simple Western system (Protein Simple). Graphs in the left column (A and B) show fold change in endogenous phosphorylated tau levels in half-brain homogenates collected at 7 d rTBI compared to sham brains using antibodies CP13 (pSer202 and pThr205, Panel A) and RZ3 (pThr231, Panel B), respectively. Graphs in the middle column (C and D) depict quantitation of phosphorylated tau as a proportion of total tau (DA9). Representative digital immunoblots of phosphorylated and corresponding total tau are depicted in the right column (E and F).

Using the same experimental conditions as in this study, we previously showed that endogenous murine tau shows a transient increase in phosphorylation that resolves to baseline 7 days after CHIMERA rTBI [34]. To determine the effect of AAS exposure, we assessed the phosphorylation levels of endogenous murine tau in half-brain homogenates collected at 7 d post-rTBI using two antibodies directed against different tau phosphorylation sites, namely CP13 (pSer202) and RZ3 (pThr231). Total murine tau levels were determined by the antibody DA9. Simple Western analysis showed that, as expected from our previous study, tau phosphorylation levels were not significantly different in the rTBI group compared to sham controls at 7 d post injury (Fig 3). AAS treatment also had no significant effect on tau phosphorylation levels at this time point (Fig 3).

### Chronic AAS Treatment Downregulates 5-HT1B Receptor Expression in the Substantia Nigra Irrespective of rTBI

**Fig 4:**
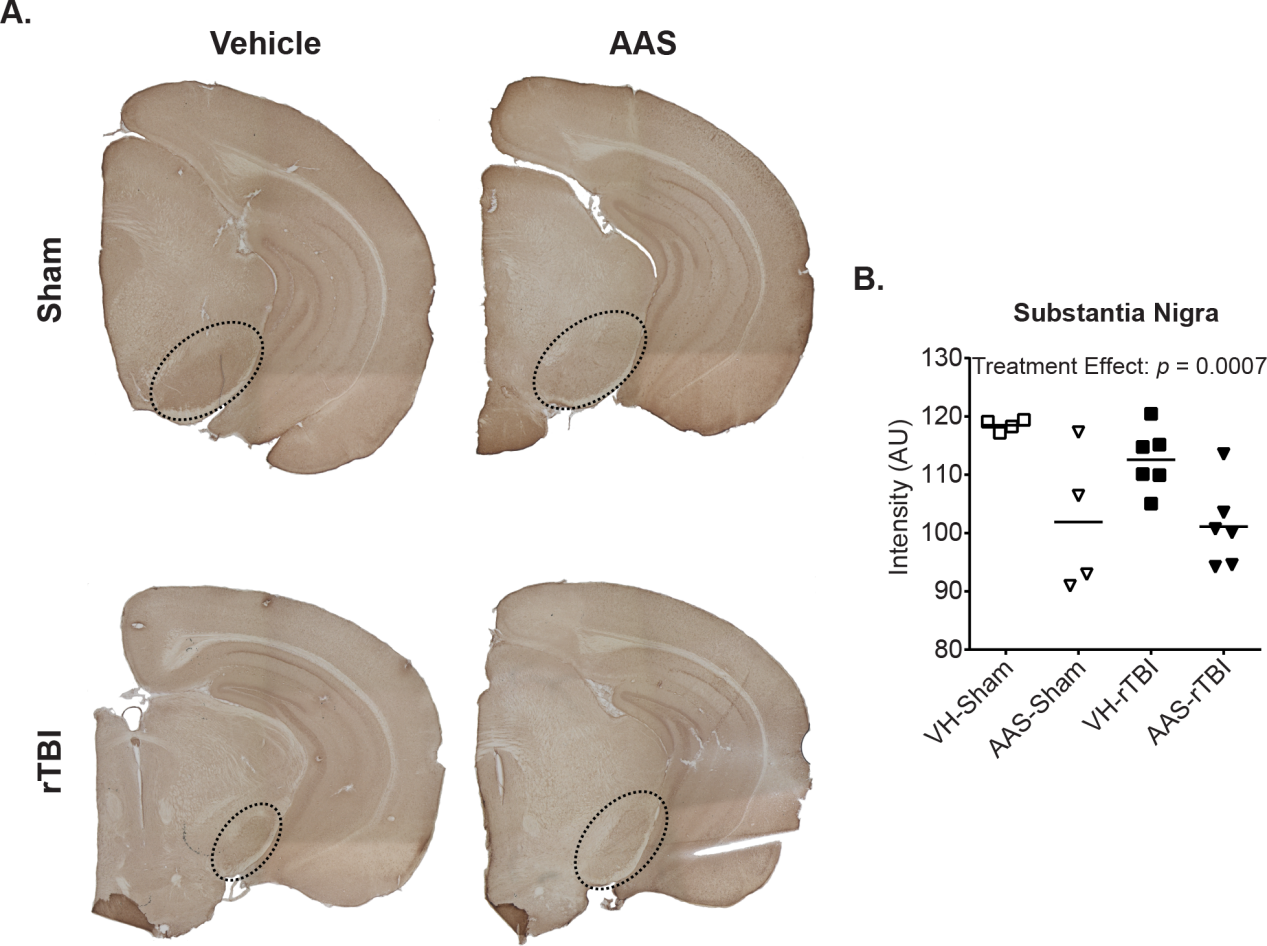
Chronic AAS treatment downregulates 5-HT1B receptor expression in the substantia nigra. Immunohistochemistry was used to assess 5-HT1B receptor expression levels. Representative images of whole-mount sections for AAS-treated and control brains are depicted in Panel A. 5-HT1B receptor expression in the substantia nigra (dashed circles) was quantified by measuring the mean grey intensity of the selected brain area on an 8-bit grayscale image. Graph in Panel B depicts mean staining intensity in arbitrary units.

Chronic AAS treatment has been reported to alter neurotransmitter systems, particularly monoamine neurotransmitters including serotonin (5-hydroxytryptmaine /5-HT) and dopamine, which are associated with mood, reward, anxiety and aggressive behavior [32]. To confirm the neurochemical effects of AAS treatment in the present study, we assessed serotonin receptor (5-HT1B) expression in brain tissues by immunohistochemistry and found significantly decreased staining intensity selectively in the substantia nigra (Fig 4, treatment effect: *p* = 0.0007). The decrease in 5-HT1B expression was independent of injury status (Fig 4B, rTBI effect: *p* = 0.3293).

### Chronic AAS Treatment Significantly Exacerbates Silver Uptake in rTBI-Damaged Axons without Affecting APP Immunostaining

**Fig 5:**
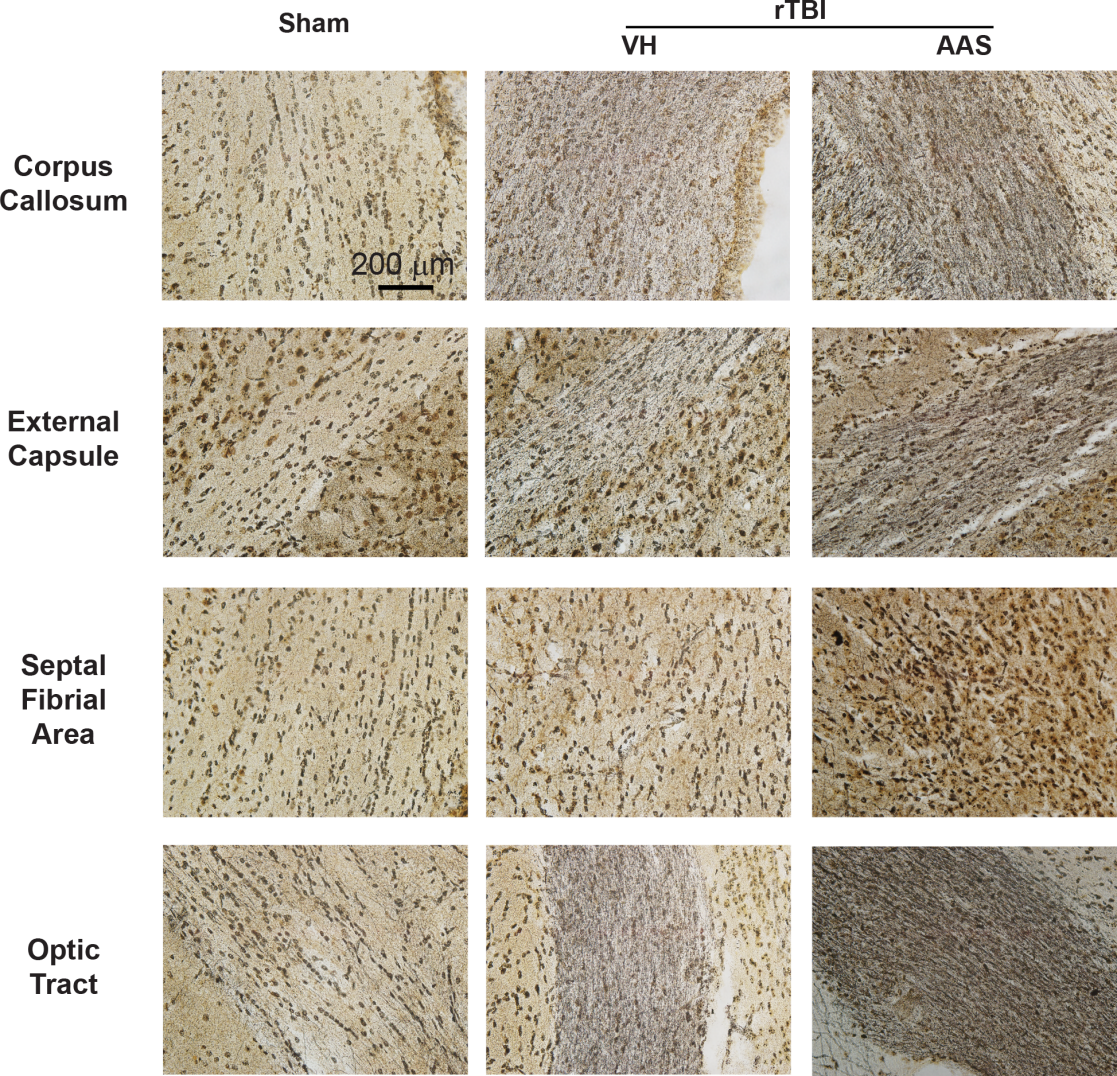
Chronic AAS treatment in mice exacerbates post-rTBI axonal injury. Post-rTBI axonal damage was assessed with silver staining. Representative 40X-magnified images of corpus callosum, external capsule, septal-fimbrial area and optic tract of sham (left column) and VH- (middle column) and AAS-treated (right column) rTBI brains are depicted.

**Fig 6:**
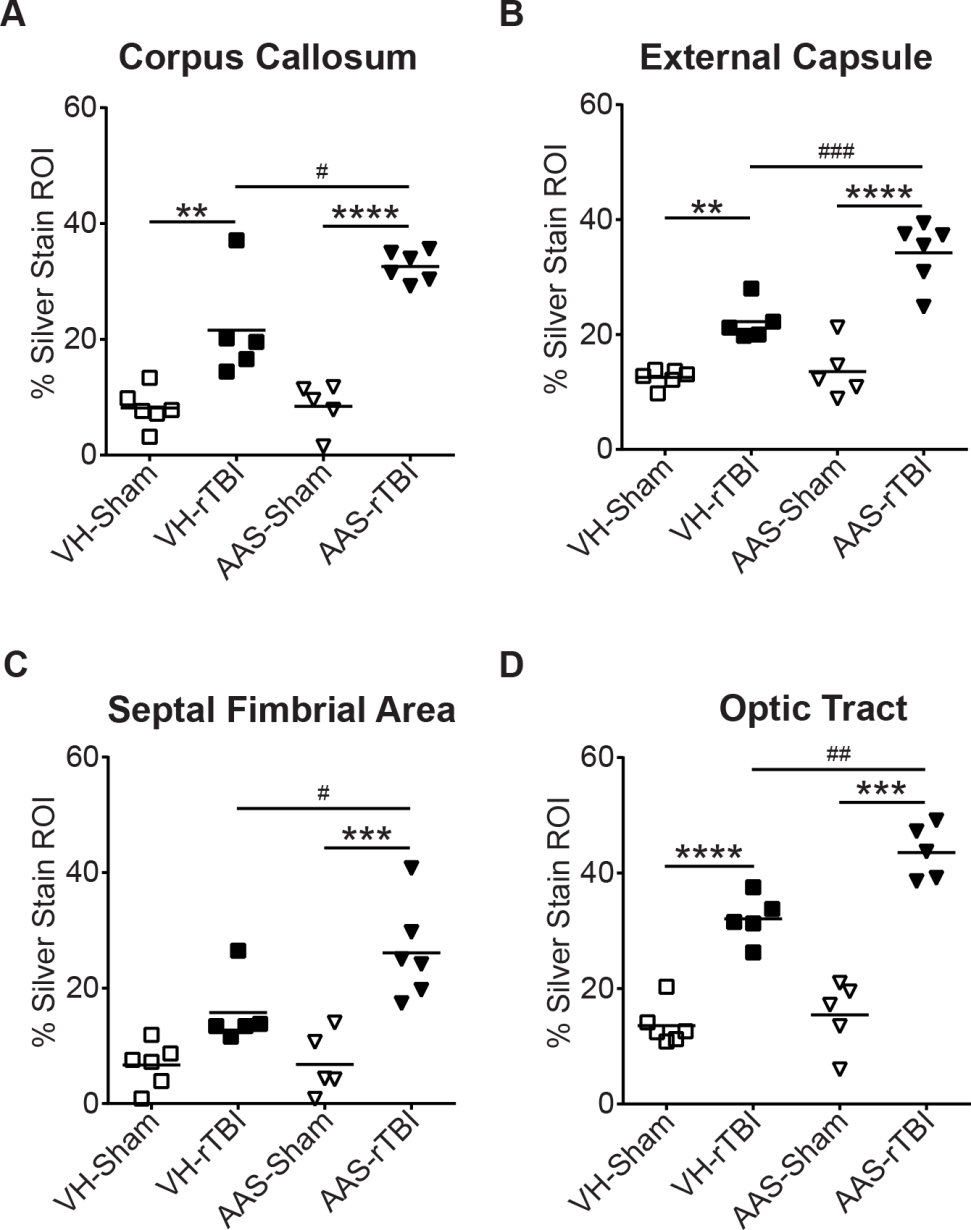
Quantitative assessment of silver stained images. Silver stained images were quantified by calculating the percent of region of interest (ROI) in the white matter tract area that was stained with silver. Graphs indicate percent of the ROI showing positive signal in the respective white matter regions. For all graphs, * indicates a significant rTBI effect compared to the respective sham values and # indicates a significant treatment effect between rTBI groups. Data are analyzed by two-way ANOVA followed by a Tukey post-hoc test. For all graphs **: *p* < 0.01, ***: *p* < 0.001, ****: *p* < 0.0001, #: *p* < 0.05, ##: *p* < 0.01, ###: *p* < 0.001.

Silver staining was used to assess post-rTBI axonal damage at 7 d post rTBI. As we have previously reported [34], CHIMERA-injured brains revealed widespread multifocal axonal injury, as indicated by intense punctate and fiber-associated argyrophilic structures in several white matter tracts including the corpus callosum, external capsule, septal-fimbrial area, and optic tract (Fig 5). Axonal injury was observed at both coup (corpus callosum) and contrecoup (optic tract) regions, indicating a diffuse injury pattern. With the exception of septal-fimbrial area in the VH-rTBI group, quantitative analysis of silver stained images revealed significantly increased silver uptake in all of the above-mentioned white matter regions for both rTBI groups (Fig 6). AAS treatment further exacerbated rTBI-induced axonal damage as indicated by a significant treatment X rTBI interaction in the corpus callosum (*p* = 0.026), external capsule (*p* = 0.005), septal-fimbrial area (*p* = 0.033) and optic tract (*p* = 0.029) (Fig 5). By contrast, neither rTBI nor AAS led to altered APP immunoreactivity under the conditions used here (S4 Fig), consistent with previous reports of increased sensitivity of silver compared with APP immunostaining in wild-type rodents with experimental TBI [35–37].

### Chronic AAS Treatment Exacerbates rTBI-Induced Microgliosis

**Fig 7:**
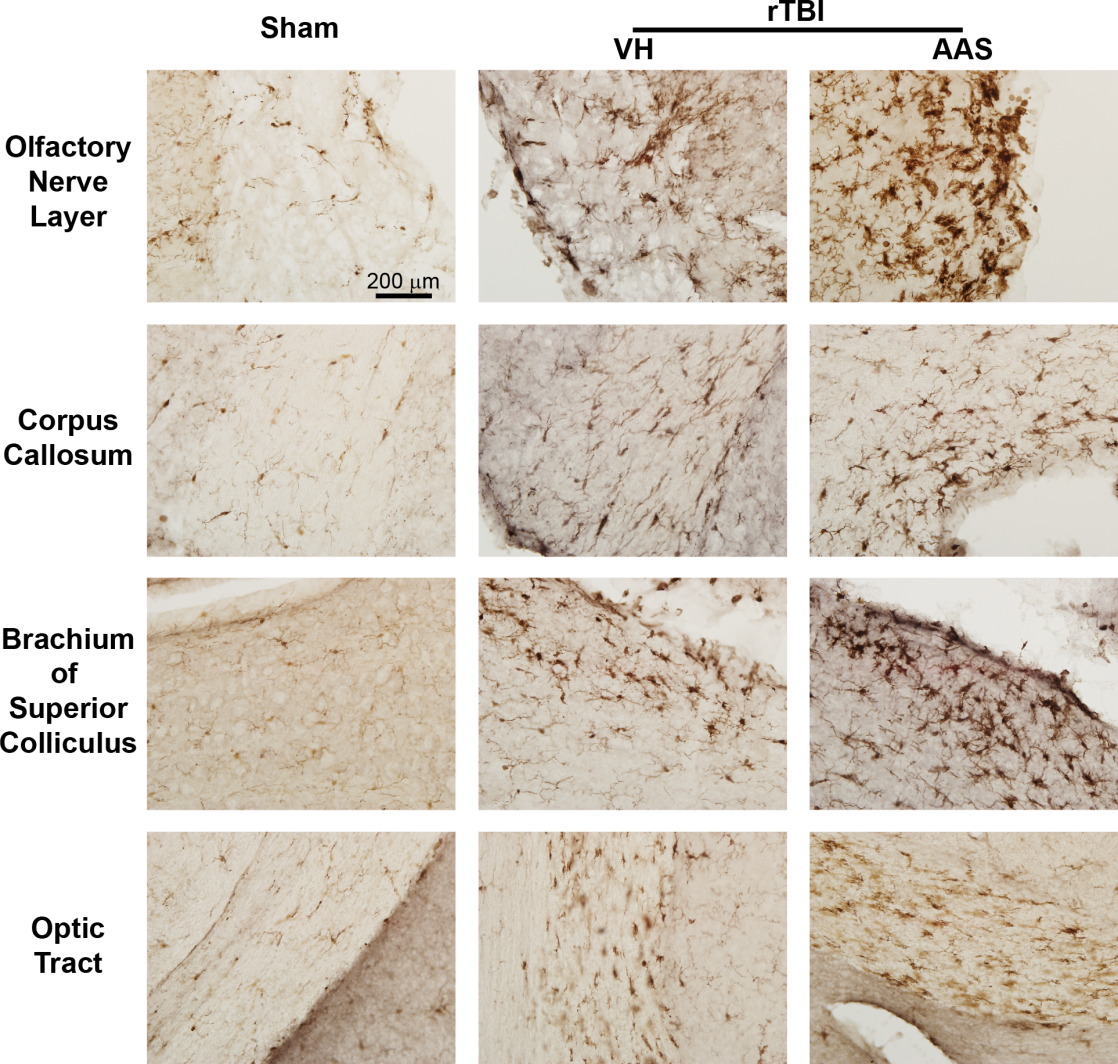
Chronic AAS treatment in mice augments post-rTBI microgliosis. Post-rTBI microglial activation was assessed with Iba1 immunohistochemistry at 7 d. Representative 40X-magnified images of white matter regions show resting microglia in sham brains (left column) and activated microglia in injured brains (second and third columns).

**Fig 8:**
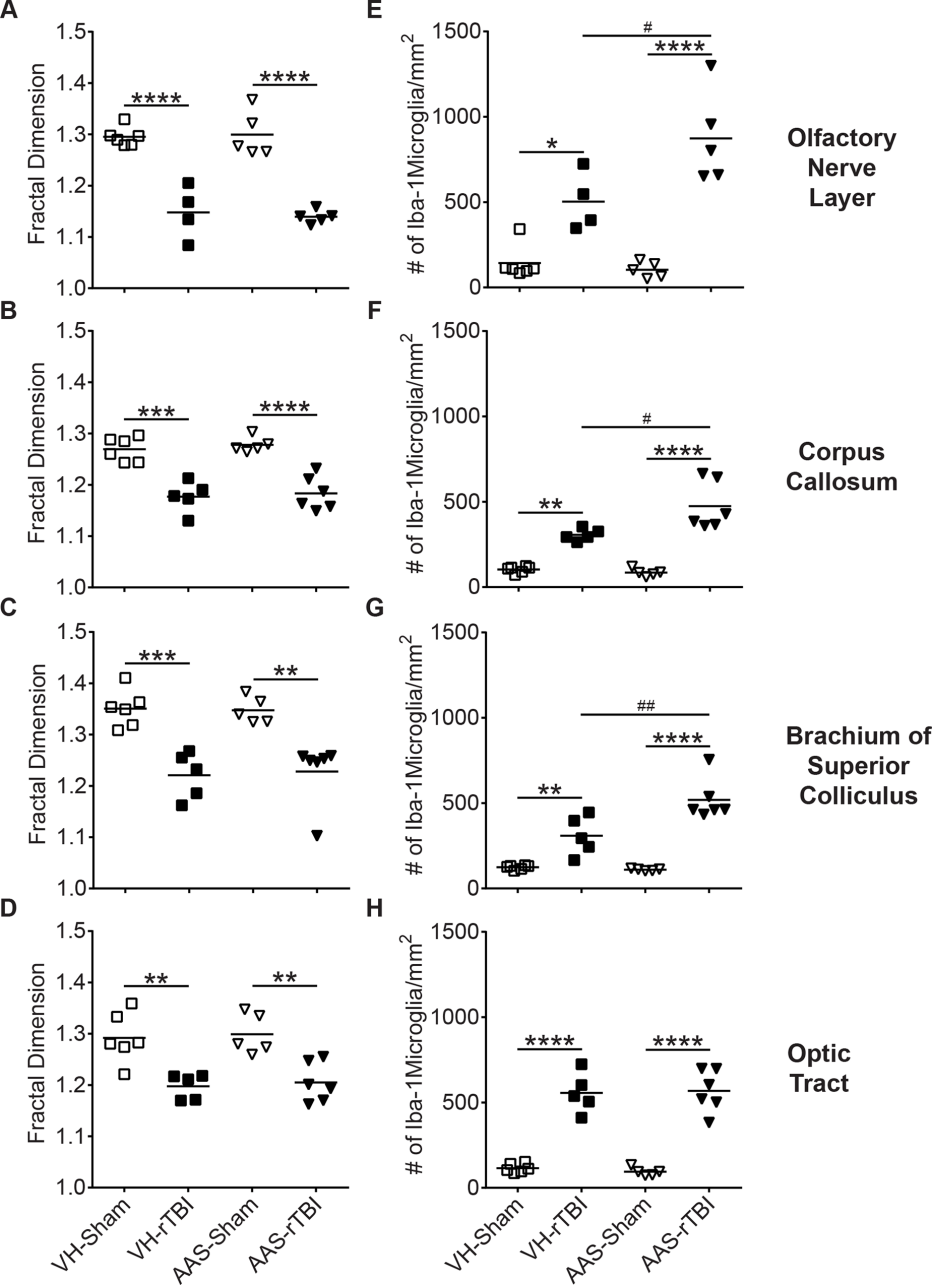
Quantitative analysis of the microglial response to rTBI. Microglial morphology was quantitatively assessed using fractal analysis. Graphs in the left column represent fractal dimension for microglial morphology in (A) olfactory nerve layer, (B) corpus callosum, (C) brachium of superior colliculus and (D) optic tract. Graphs in the right column (E-H) show number of Iba1-positive cells per mm^2^ in the same white matter regions. For all graphs, * indicates a significant rTBI effect compared to the respective sham values and # indicates a significant treatment effect between rTBI groups. Data are analyzed by two-way ANOVA followed by a Tukey post-hoc test. For all graphs *: *p* < 0.05, **: *p* < 0.01, ***: *p* < 0.001, ****: *p* < 0.0001, #: *p* < 0.05, ##: *p* < 0.01.

Using Iba1 immunohistochemistry, we observed significant microglial activation throughout several white matter regions including the olfactory nerve layer, corpus callosum, brachium of superior colliculus and optic tract of injured brains compared to sham controls (Fig 7). Microglia in sham brains displayed high fractal dimensions consisting of highly complex, extensively branched and ramified morphology indicative of the resting state (Fig 8A–8D). By contrast, microglia in injured brains from both VH and AAS-treated groups showed significantly reduced fractal dimensions in the above white matter regions indicative of an activated state (Fig 8A–8D). AAS treatment did not alter microglial morphology in the absence of injury (Fig 8A–8D). In addition to the changes in microglial morphology, VH-rTBI and AAS-rTBI groups showed significant increases in the number of Iba-1 stained microglia in the same white matter regions (Fig 8E–8H) including the olfactory nerve layer (Fig 8E), corpus callosum (Fig 8F), brachium of superior colliculus (Fig 8G) and optic tract (Fig 8H) (rTBI effect for all regions: *p* < 0.0001), indicating that injury induced proliferation and/or recruitment of immune cells. Intriguingly, except for the optic tract, brains in the AAS-rTBI group showed significantly higher numbers of Iba1-positive microglia in the olfactory nerve layer (Fig 8E, Treatment X rTBI interaction: *p* = 0.014), corpus callosum (Fig 8F, Treatment X rTBI interaction: *p* = 0.012), brachium of superior colliculus (Fig 8G, Treatment X rTBI interaction: *p* = 0.006) compared to the injured brains in the VH treatment group, demonstrating an exacerbated inflammatory reaction in AAS-treated injured mice.

## Discussion

The main objective of this study was to determine whether antecedent chronic AAS exposure at supra-physiological doses alters behavioral deficits and/or neuropathology during the acute period following rTBI. We treated wild-type mice with a cocktail of three commonly used AAS, representative of a high-dose regimen in humans, for seven weeks prior to inducing two consecutive mild TBIs spaced 24 h apart using our CHIMERA model of closed-head impact-acceleration TBI. Mice were followed behaviorally for 7 d after rTBI during which AAS treatment was continued. Prior to rTBI, AAS-treated mice exhibited the expected morphological changes in body, seminal vesicle and testicular weights, as well as the expected increase in aggressive behavior. In the acute period of 7 d after rTBI, AAS-treated mice displayed more severe axonal injury and significantly increased microgliosis in several white matter tracts, but did not exhibit significantly worsened behavioral deficits or sustained phosphorylation of murine tau under the experimental conditions used here. Future studies will be needed to determine if more severe injury, a greater cumulative number of injuries, or additional time points of analyses unveil significant behavioral outcomes between AAS-treated and control mice after rTBI. In addition, studies using transgenic mice will be needed to investigate whether AAS exposure influences additional neuropathological changes including tau-containing neurofibrillary tangles.

To our knowledge, only one other preclinical study on chronic AAS effects after TBI has been reported. Using the Marmarou method of dropping a weight onto a metal disk affixed to the exposed skull of rats, Mills et al reported no difference in axonal injury as measured by APP immunohistochemistry when analyzed at 30 d after TBI [33]. No other outcome was reported. Our study has several important differences compared to Mills et al that could explain why we observed exacerbated axonal pathology and inflammation whereas Mills et al did not. Compared to the Marmarou weight drop model, CHIMERA is nonsurgical and allows completely free and reliable head motion after impact, thus recapitulating the biomechanical responses of the head after impact in humans [34]. Head motion has previously been shown to be an important parameter of injury in blast TBI [38] and is believed to be a major contributor to the biomechanical strain that underlies DAI [39]. That CHIMERA uses only isoflurane anesthesia for a short duration (~ 4 min) may also allow neuropathological pathways to be initiated that could be suppressed by a combination of anesthetic and analgesic agents required for surgical TBI models. Additionally, many weight-drop TBI models are susceptible to large experimental variability [40]. The post-rTBI behavioral and neuropathological assessments data from the present study are in close agreement with our previously-reported observation [34] indicating excellent reproducibility of CHIMERA model. We treated animals with a cocktail of three most-commonly used AAS to simulate a “stacking” regimen typically used by athletes to increase the potency of each drug [28], whereas Mills et al used nandrolone only. We assessed animals after 8 weeks of AAS exposure and 7 d post rTBI, whereas Mills et al used a 12 week nandrolone treatment duration and assessed animals at 30 d post-TBI. Our study was specifically designed to correspond roughly to adolescence/young adulthood in mice at the time of rTBI, whereas Mills et al used adult rats. We confirmed that AAS treatment produced the expected physiological effects and also demonstrated decreased 5-HT1B receptor expression in the substantia nigra. Serotonin (5-HT) is well-studied neurotransmitter that is consistently shown as an inhibitor of aggression [41, 42]. A lower level of 5-HT or its metabolites, or impaired 5-HT receptor (5-HT1A and 5-HT1B) functions is linked to aggressive behavior in clinical and animal studies [43–45]. On the other hand, 5-HT receptor agonists reduce aggression in experimental settings [46, 47]. Several studies have shown that chronic AAS exposure reduces the levels of serotonin and its metabolites [48, 49], 5-HT-immunoreactive neuronal fibers [50] as well as receptor expression [51, 52]. Regionally, the substantia nigra has the highest 5-HT1B receptor expression [53, 54].

We previously reported that CHIMERA rTBI alters LRR, NSS, as well as motor and cognitive performance [34]. While AAS treatment did not significantly exacerbate LRR, NSS or motor deficits in injured mice, we did observe a trend towards increased motor deficits in AAS-rTBI mice compared to VH-rTBI controls upon component analysis of the NSS. These observations suggest that AAS exposure may cause subtle changes in post-rTBI motor deficits during the acute phase of TBI that may or may not resolve over a further period of recovery. We did not evaluate the effect of AAS exposure on cognitive outcomes, which are not feasible to examine during this very acute phase post rTBI. Future studies will be needed to understand how AAS exposure may affect the trajectory of post-TBI behavioral deficits over the long term.

Using silver staining, we observed increased argyrophilic fibers and punctate structures in several white matter tracts throughout the brain that were significantly exacerbated by chronic AAS exposure when assessed at 7 d post-rTBI. By contrast, Mills et al examined axonal injury only in the corticospinal tract using APP immunohistochemistry 30 d after TBI. Additional studies designed to characterize dynamic changes in multiple markers of axonal damage during the days and weeks post-TBI will be an important avenue of future investigation.

We did not observe changes in the levels of phosphorylated murine tau in either the AAS-rTBI or VH-rTBI groups levels at 7 days post-rTBI, consistent with our previous observation that murine tau hyperphosphorylation levels peak between 6 h to 2 days and return to baseline levels by 7 days using the same CHIMERA injury settings used here [34]. Importantly, the anatomical differences between the human and murine brain [55], as well as species differences in tau expression [56–59], poses challenges toward replicating classical CTE tau neuropathology in wild-type mice [60].

In agreement with our previous observations [34], we observed significant widespread microgliosis in several white matter regions of injured brains. While AAS treatment alone in sham brains did not alter the microglial response, AAS treatment in combination with TBI further augmented microgliosis, which is contrary to the immunosuppressive effects of AAS in the periphery when administered at supraphysiological doses [61]. Further studies will be needed to characterize the contributions of central compared to peripheral effects of AAS on neuroinflammation, which can be affected by resident microglia as well as infiltrating macrophages [62].

An increased recognition of the potentially deleterious consequences of youth concussions has grown steadily over the past decade. Importantly, our understanding of concussion pathogenesis and resolution is yet at its infancy. Our results suggest that exposure to AAS exacerbates axonal pathology as well as microgliosis even after two relatively mild TBIs. These results could have significant implications for clinical TBI studies in athletes, as surveys indicate that 4–12% of adolescent and young adult athletes take AAS even when the established risks of these drugs are presented to them [63].

As we saw a detrimental outcome following both AAS and TBI, our work opens up additional lines of investigation. For example, we assessed post-rTBI outcomes only at a single acute post-TBI time point and under specific experimental conditions of two consecutive TBIs spaced 24 apart induced with a defined impact energy that mimics mild TBI. Future studies could explore the dynamics of behavioral and neuropathological outcomes over a longer post-rTBI period, and also investigate how injury severity and cumulative exposure may each affect outcomes in both untreated and AAS-treated animals. Since we performed TBI in week 7 of an 8 week AAS treatment regimen, our conclusions are limited to concurrent AAS use. Further studies will be needed to determine if the adverse effects of AAS on axonal integrity and neuroinflammation persist after AAS discontinuation. Additional studies will also be needed to determine the effect of AAS on deposition of tau, amyloid, and TDP-43, as the wild-type mice used in our study do not develop these neuropathologies. The present study was conducted in males only. Epidemiological data suggest that female athletes may be at greater risk for sustaining a concussion compared to males [64]. As the global prevalence rate of AAS use by female athletes is ~ 2% [29], how AAS exposure alters concussion risk in female athletes will need further investigation.

In conclusion, our study shows that AAS exposure exacerbates axonal damage and neuroinflammation after concussion, adding to the list of disincentives that could be used to reduce AAS use.

## Materials and Methods

### Animals

Gonadally-intact, 8-week old male C57BL/6 mice were randomly assigned to one of two groups, resident and intruder, prior to the start of AAS treatment. All mice were housed under a reversed 12 h light-12 h dark cycle for at least 10 d before the initiation of AAS treatment. For the duration of the study, resident mice were singly housed to facilitate inter-male aggression testing. Intruder mice were group-housed with 2–4 animals per cage and only used for resident-intruder testing (RIT, described below) at weeks 5, 6, and 7 following initiation of treatment. All animal procedures were approved by the University of British Columbia Committee on Animal Care and were performed in strict accordance with the Canadian Council on Animal Care guidelines.

### Drugs

Testosterone cypionate, 17α-methyltestosterone, and nandrolone decanoate were purchased from Steraloids (Newport, RI). Sesame oil vehicle was purchased from Sigma. A fresh cocktail of all the three AAS in sesame oil vehicle was made every week.

### Pre-rTBI AAS Administration

Resident mice were randomized into one of four treatment arms: 1) Seven-week sesame oil vehicle treatment followed by sham injury [VH-Sham, N = 9], 2) seven-week AAS cocktail treatment followed by sham injury [AAS-Sham, N = 9], 3) seven-week sesame oil vehicle treatment followed by rTBI [VH-rTBI, N = 12], and 4) seven-week AAS treatment followed by rTBI [AAS-rTBI, N = 12]. Mice in the two AAS treatment arms received a cocktail of three AAS (2.5 mg/kg/day each of testosterone cypionate, 17α-methyltestosterone, and nandrolone decanoate) for a total dose of 7.5 mg/kg/day via subcutaneous injection starting from 8 week of age [65, 66]. This daily dose of AAS is equivalent to typical high-dose AAS regimens reported to be used by athletes [65]. Mice in the two VH treatment arms received an equivalent volume of sesame oil vehicle (~ 150 μl/day) via subcutaneous injection. The drugs were administered as a single daily dose for 5 d/week from 8–16 weeks of age (S5A Fig). To avoid excessive fluctuations in plasma drug concentration, as well as to simulate the “cycling” pattern of AAS administration by athletes, we designed a typical weekly injection schedule as follows: Day 1–3 injections, Day 4 no injection, Day 5–6 injections, Day 7 no injection (S5B Fig). Drug treatment was maintained throughout the entire experiment until tissues were harvested 7 d following rTBI. In addition, for behavioral comparisons, we included two age-matched naïve treatment groups that underwent either sham (N = 6) or rTBI (N = 10) procedure but received neither vehicle/AAS treatment nor the handling associated with the weekly injections.

### CHIMERA rTBI

At the end of the 7^th^ week of AAS treatment, mice underwent rTBI and sham procedures using CHIMERA as described [34]. Briefly, animals received two successive closed-head impacts spaced 24 h apart under isoflurane anesthesia using a pneumatically-driven 50 g steel piston calibrated to deliver 0.5 J of kinetic energy at the point of impact. Sham animals underwent all procedures except for impact. Animals were followed for 7 d after the second TBI with behavioral tests followed by tissue collection on day 7 as described below.

### Behavioral Analyses

Duration of loss of righting reflex (LRR) following each of the two closed-head impacts was recorded as described [34]. Neurological impairment was assessed using the neurological severity score (NSS) at 1 h and at 1, 2, and 7 d post-rTBI as described.[34] Motor function was assessed using the accelerating rotarod test at 1, 2, and 6 d post-rTBI as described [34, 67]. Open field activity and thigmotactic behavior were assessed at 1 and 6 d after the second TBI as described previously [34].

AAS-induced aggressive behavior in resident mice was assessed using the RIT [66] in weeks 5 and 6 (pre-rTBI) of treatment and on the 5^th^ day after the second TBI. In each week, a single RIT session was conducted starting at the beginning of the dark phase of the12 h-12 h dark-light cycle in the home cage of resident mice. All bedding material in the resident mice cages was kept unchanged for 7 d prior to each RIT to maintain scents associated with territorialization. Immediately prior to each test, nesting material from the resident’s home cage was removed to prevent visual obstruction during videotaping. Following a 5 min acclimation period, a weight-matched male intruder mouse was introduced to the resident’s home cage and a timer was started. Resident-intruder interactions were videotaped for a maximum duration of 10 min. Upon the first evidence of a fight between the pair within 10 min, the timer was stopped and the mice were immediately separated. At the end of each RIT session, resident mice were transferred to a new cage and intruder mice were returned to their home cage. Intruder mice were identified with distinctive tail marks made with a non-toxic marker. Resident-intruder pairs were kept constant between tests. Recorded sessions were later evaluated by an observer blinded to the treatment and injury status for recording latency to fight.

### Tissue Collection

Tissues were collected at 7 d post-rTBI as described previously [34, 67]. Briefly, ketamine-xylazine-anesthetized animals were perfused with ice-cold heparinized phosphate-buffered saline (PBS). Brain, seminal vesicles and testes were collected, weighed and photographed for morphological analysis. The brain was longitudinally bisected and hemisections were processed for histology and biochemistry as described [34, 67].

### Quantitative Assessment of Phosphorylated Tau, 5-HT1B Receptor Expression, Axonal Damage and Microglial Activation

Phosphorylated and total tau in half-brain RIPA lysates were assessed using the Protein Simple Wes apparatus as described [34].

Expression of serotonin receptor 1 subtype B (5-HT1B) was assessed with immunohistochemistry. Briefly, 3–4 coronal brain sections (40 μm thick) coronal brain sections were treated with 0.3% hydrogen peroxide for 10 min and blocked with 3% normal goat serum (NGS) in PBS for 1 h. Sections were incubated overnight with rabbit anti-mouse 5-HT1B receptor antibody (Abcam, Ab13896, 1:300) in 3% NGS. After washing in PBS the sections were incubated with a biotinylated goat anti-rabbit secondary antibody (1:1000 in PBS) for 1 h. Sections were visualized with horseradish peroxidase (Vectastain Elite ABC Kit PK-6120, Vector Laboratories (Canada) Inc, Burlington, ON) and DAB substrate. From each section, 5X-magnified images were captured with a Zeiss microscope (Axio Observer Z1) using Axiocam 150 color camera and stitched together with Zen Pro imaging software (version 2). For quantification of 5-HT1B expression in the substantia nigra, images were first converted to 8-bit grayscale image (Min:0, Max: 256) and the mean grey intensity of the region of interest was quantified using ImageJ (version 1.48, NIH).

Post-rTBI axonal pathology was assessed with silver staining and APP immunohistochemistry. Silver staining was performed using FD Neurosilver kit (PK301A, FD NeuroTechnologies, Inc, Columbia, MD) as described [34]. For APP immunohistochemistry, 3–4 coronal sections were washed with PBS and treated with 1% hydrogen peroxide. Antigen retrieval was carried out for 8 min in a microwave pressure cooker using 100mM Tris and 50 mM EDTA buffer (pH 8.0) followed by blocking with mouse IgG blocking reagent (M.O.M. Basic Immunodetection Kit, BMK-2202, Vector Laboratories, Burlingame, CA) according to the manufacturer’s instructions. The sections were incubated for 45 min at room temperature with murine APP antibody (clone 22C11, 1:5000, EMD Millipore Corporation). After washing with PBS, the sections were incubated for 10 min at room temperature with biotinylated anti-mouse secondary antibody (M.O.M. Basic Immunodetection Kit, BMK-2202, Vector Laboratories, Burlingame, CA) according to the manufacturer’s instructions. Sections were visualized with horseradish peroxidase (Vectastain Elite ABC Kit PK-6120, Vector Laboratories (Canada) Inc, Burlington, ON) and DAB substrate and counterstained with hematoxylin. From each section 20X-magnified white matter regions of interest were imaged with a Zeiss microscope (Axio Observer Z1) using Axiocam 150 color camera.

Microglial activation was assessed with Iba1 immunohistochemistry as described [34, 67]. Microglial morphology was quantified using fractal analysis and cell counting whereas silver staining was assessed by quantifying percent stained area of the region of interest of white matter using ImageJ (version 1.48, NIH) as described [34].

### Statistical Analyses

Body weight data were analyzed with repeated measures two-way ANOVA with followed by a Bonferroni post hoc test. Tissue weight data were analyzed using two-tailed unpaired t test. All behavioral data were analyzed for rTBI, time and treatment effects as well as rTBI X time, rTBI X treatment and rTBI X time X treatment interactions using a repeated measures general linear model using SPSS (v 20). Histology and biochemistry data were analyzed for rTBI, treatment and rTBI X treatment effects using two-way ANOVA with a Tukey post-hoc test using GraphPad Prism (v 6.05). For all the above statistical analyses, a *p* value of < 0.05 was considered significant.

## Supporting Information

S1 Fig Percent of mice failing on beam walk tests. Beam walk tests are an integral component of NSS testing where mice are assessed for their ability to successfully walk on 3 cm, 2 cm and 1 cm-wide beams representing increasing task difficulty. Graphs represent the percentage of mice in each of the four study arms failing on each of the three beams.
(TIF)

S2 Fig CHIMERA rTBI does not significantly affect general mobility. General mobility was tested by the open field test at 1 and 6 d post-injury. There was no rTBI effect at any time point for the total distance travelled (A) and time spent immobile (B). Data are presented as the mean ± SEM and analyzed by repeated measures general linear model. Legends are consistent across all graphs.
(TIF)

S3 Fig Vehicle treatment does not significantly affect post-rTBI behavior. To test whether the handing and injections associate with VH treatment itself altered post-rTBI behavior, we compared naïve mice with the respected VH-treatment groups. Data in the graphs are presented as mean ± SEM values. Legends are consistent across all graphs.
(TIF)

S4 Fig Neither rTBI nor AAS induces APP accumulation in damaged axons. Post-rTBI axonal injury was assessed with APP immunohistochemistry. Representative 20X-magnified images of corpus callosum, external capsule, and optic tract of sham (left column) and VH- (middle column) and AAS-treated (right column) rTBI brains are depicted.
(TIF)

S5 Fig Experimental design. (A) Schematic details of AAS treatment, rTBI and post-rTBI assessment. AAS: androgenic-anabolic steroid cocktail, LRR: loss of righting reflex, NSS: neurological severity score, OF: open field behavior, RIT: resident-intruder test, RR: rotarod, VH: sesame oil vehicle. (B) Example of a weekly AAS or VH injection schedule used in the present study.
(TIF)

S1 Raw Data The raw data captured during the present study are compiled into a single Excel spreadsheet. Individual assay data are presented under individual tabs.
(XLSX)

## Abbreviations

AAS: androgenic-anabolic steroid
APP: amyloid precursor protein
CHIMERA: closed-head impact model of rotational engineered acceleration
CTE: chronic traumatic encephalopathy
DAI: diffuse axonal injury
LRR: loss of righting reflex
mTBI: mild traumatic brain injury
NSS: neurological severity score
PCS: post-concussion syndrome
PTS: posttraumatic symptoms
RIT: resident-intruder test
rTBI: repetitive traumatic brain injury
SRC: sport related concussion
TBI: traumatic brain injury
VH: sesame oil vehicle
