# Supplementary figures and images for: Chronic Exposure to Androgenic-Anabolic Steroids Exacerbates Axonal Injury and Microgliosis in the CHIMERA Mouse Model of Repetitive Concussion
Source: PLoS One. 2016 Jan 19;11(1):e0146540. doi: 10.1371/journal.pone.0146540 (PMC4718534; doi:10.1371/journal.pone.0146540)

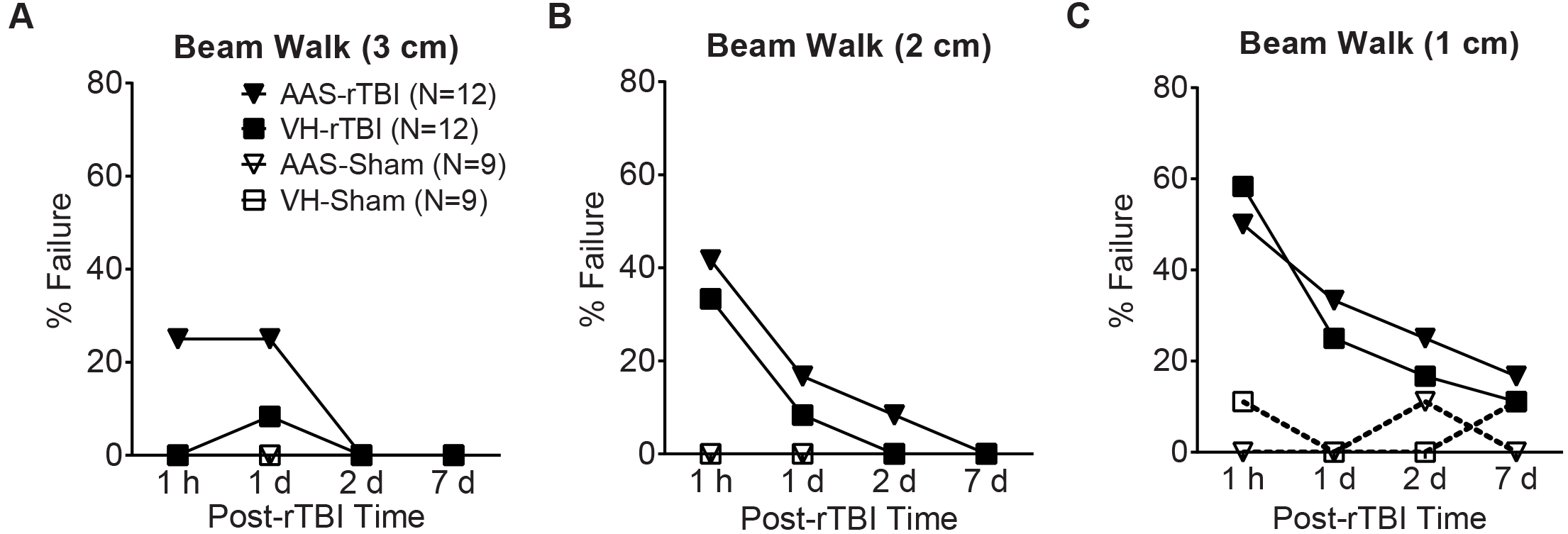

Supplement: S1 Fig — Beam walk tests are an integral component of NSS testing where mice are assessed for their ability to successfully walk on 3 cm, 2 cm and 1 cm-wide beams representing increasing task difficulty. Graphs represent the percentage of mice in each of the four study arms failing on each of the three beams. (TIF) [file pone.0146540.s001.tif]

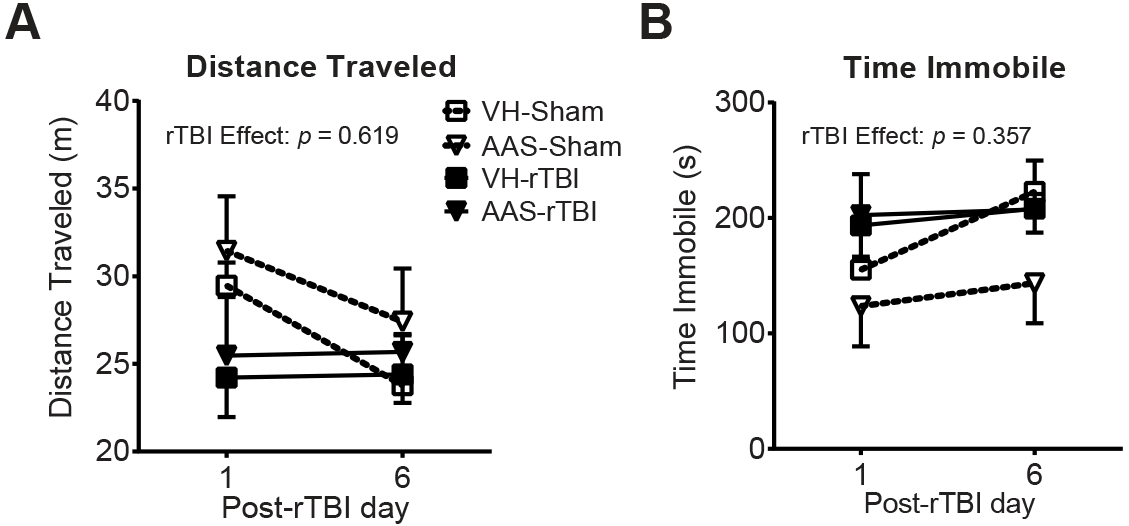

Supplement: S2 Fig — General mobility was tested by the open field test at 1 and 6 d post-injury. There was no rTBI effect at any time point for the total distance travelled (A) and time spent immobile (B). Data are presented as the mean ± SEM and analyzed by repeated measures general linear model. Legends are consistent across all graphs. (TIF) [file pone.0146540.s002.tif]

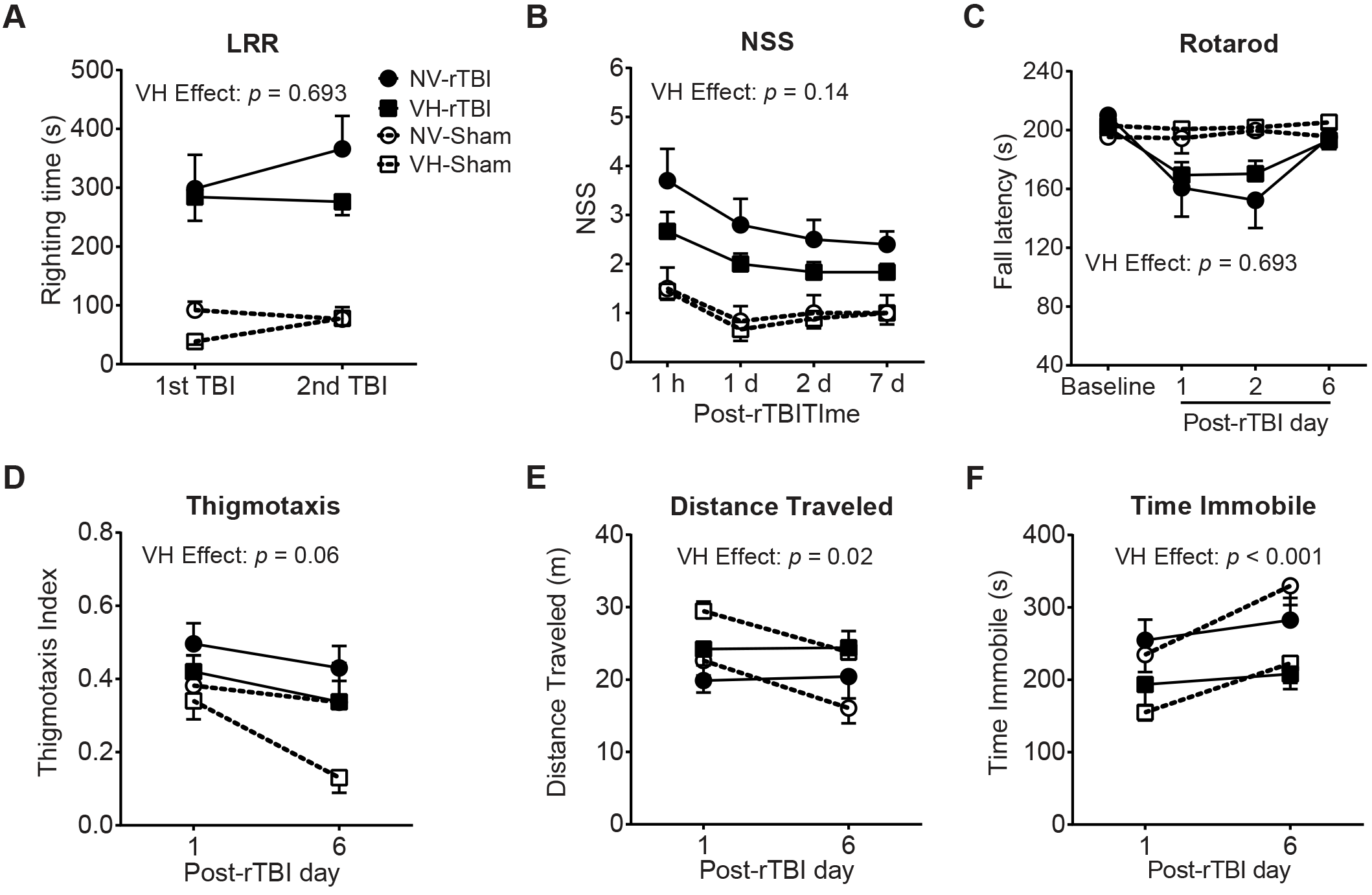

Supplement: S3 Fig — To test whether the handing and injections associate with VH treatment itself altered post-rTBI behavior, we compared naïve mice with the respected VH-treatment groups. Data in the graphs are presented as mean ± SEM values. Legends are consistent across all graphs. (TIF) [file pone.0146540.s003.tif]

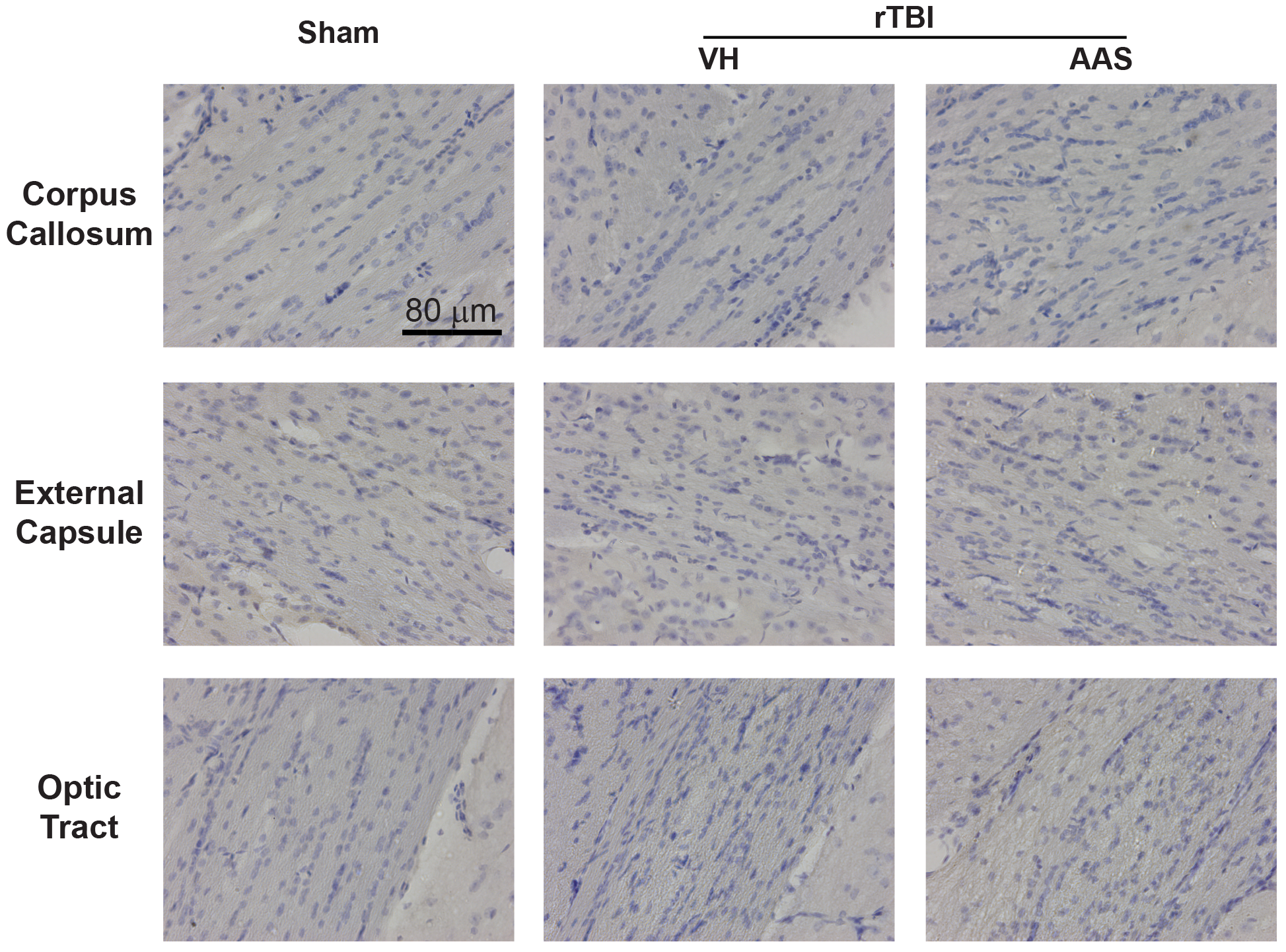

Supplement: S4 Fig — Post-rTBI axonal injury was assessed with APP immunohistochemistry. Representative 20X-magnified images of corpus callosum, external capsule, and optic tract of sham (left column) and VH- (middle column) and AAS-treated (right column) rTBI brains are depicted. (TIF) [file pone.0146540.s004.tif]

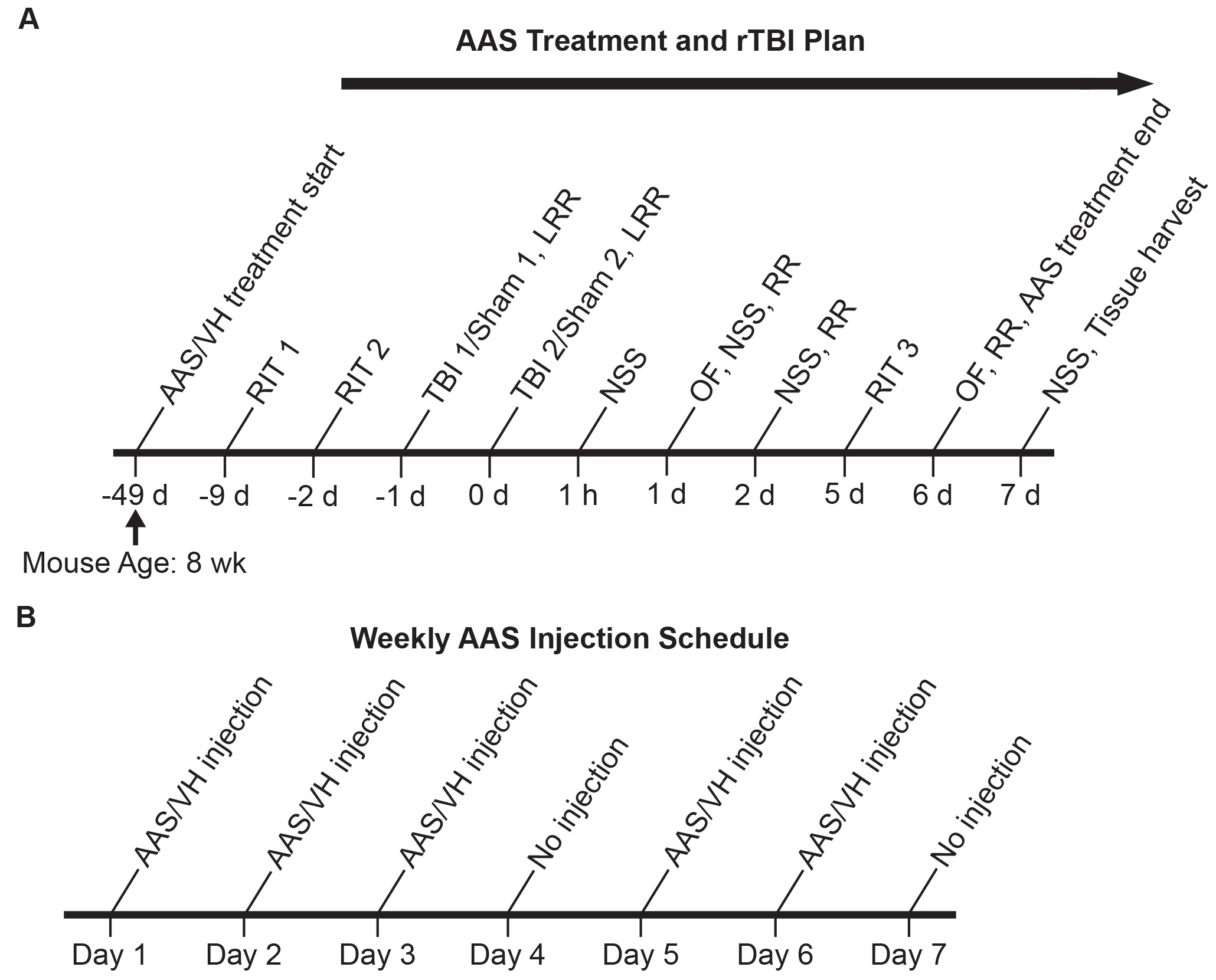

Supplement: S5 Fig — (A) Schematic details of AAS treatment, rTBI and post-rTBI assessment. AAS: androgenic-anabolic steroid cocktail, LRR: loss of righting reflex, NSS: neurological severity score, OF: open field behavior, RIT: resident-intruder test, RR: rotarod, VH: sesame oil vehicle. (B) Example of a weekly AAS or VH injection schedule used in the present study. (TIF) [file pone.0146540.s005.tif]
